# Network Profiles of the Dorsal Anterior Cingulate and Dorsal Prefrontal Cortex in Schizophrenia During Hippocampal-Based Associative Memory

**DOI:** 10.3389/fnsys.2016.00032

**Published:** 2016-04-07

**Authors:** Eric A. Woodcock, Sunali Wadehra, Vaibhav A. Diwadkar

**Affiliations:** ^1^Department of Psychiatry and Behavioral Neurosciences, Wayne State University School of MedicineDetroit, MI, USA; ^2^Translational Neuroscience Program, Wayne State University School of MedicineDetroit, MI, USA

**Keywords:** schizophrenia, neural network dysfunction, associative memory, cognitive control, psychophysiological interaction analyses

## Abstract

Schizophrenia is a disorder characterized by brain network dysfunction, particularly during behavioral tasks that depend on frontal and hippocampal mechanisms. Here, we investigated network profiles of the regions of the frontal cortex during memory encoding and retrieval, phases of processing essential to associative memory. Schizophrenia patients (*n* = 12) and healthy control (HC) subjects (*n* = 10) participated in an established object-location associative memory paradigm that drives frontal-hippocampal interactions. Network profiles were modeled of both the dorsal prefrontal (dPFC) and the dorsal anterior cingulate cortex (dACC) as seeds using psychophysiological interaction analyses, a robust framework for investigating seed-based connectivity in specific task contexts. The choice of seeds was motivated by previous evidence of involvement of these regions during associative memory. Differences between patients and controls were evaluated using second-level analyses of variance (ANOVA) with seed (dPFC vs. dACC), group (patients vs. controls), and memory process (encoding and retrieval) as factors. Patients showed a pattern of exaggerated modulation by each of the dACC and the dPFC during memory encoding and retrieval. Furthermore, group by memory process interactions were observed within regions of the hippocampus. In schizophrenia patients, relatively diminished modulation during encoding was associated with increased modulation during retrieval. These results suggest a pattern of complex dysfunctional network signatures of critical forebrain regions in schizophrenia. Evidence of dysfunctional frontal-medial temporal lobe network signatures in schizophrenia is consistent with the illness’ characterization as a disconnection syndrome.

## Introduction

Schizophrenia is a debilitating psychiatric disorder affecting 1–2% of people worldwide (Schultz and Andreasen, 1999). The illness is characterized by cognitive dysfunction (Elvevåg and Goldberg, 2000; Fioravanti et al., 2005), with learning and memory deficits particularly evident (Aleman et al., 1999; Diwadkar et al., 2008). These deficits are associated with impairments in frontal, cingulate, and hippocampal function, and dysfunctional brain network interactions within these circuits (Stephan et al., 2006; Lewis and González-Burgos, 2008; Bányai et al., 2011; Wadehra et al., 2013).

Associative memory is a psychological domain of clinical relevance that is ideally suited to probe network dysfunction in schizophrenia patients. Associative memories and associative learning involve the *encoding* of memoranda such that the paired memory trace is available for subsequent *retrieval* (Jackson and Schacter, 2004; Miller and D’Esposito, 2012). Patterns of brain network interactions during distinct periods of memory *encoding* and memory *retrieval* collectively sub serve associative memory proficiency. This proficiency has been related to the hippocampus’ role in binding (Izquierdo and Medina, 1997) via mechanisms of synaptic plasticity (Stephan et al., 2006), and at the network scale, is underpinned by frontal-hippocampal network interactions (Simons and Spiers, 2003). Moreover, tasks that probe associative memory frequently provide excellent temporal signatures of behavior, that is well characterized by negatively accelerated learning functions (Bányai et al., 2011; Woodcock et al., 2015). Finally, associative memory and learning provides a general translational framework linking molecular mechanisms of memory with macroscopic systems, as these domains have been linked both to the brain’s dopaminergic (Howes and Kapur, 2009) and glutamatergic systems (Konradi and Heckers, 2003). Notably, both dopamine and glutamate are implicated in the pathophysiology of schizophrenia (Goldman-Rakic, 1999; Stephan et al., 2006).

Although their network bases are not altogether clear, fMRI studies (targeting brain function at the macroscopic systems level) have elucidated patterns of cortical-hippocampal dysfunction and impaired associative memory in schizophrenia. First, patients exhibit exaggerated activation (Wadehra et al., 2013) in multiple regions including the dorsal prefrontal cortex (dPFC), hippocampus, and basal ganglia. In addition, patients perform less proficiently than controls on associative memory tasks showing reduced capacity for learning associations over time (Diwadkar et al., 2008; Wadehra et al., 2013). *Unsurprisingly, these deficits have been related to impaired cognitive control mechanisms in brain networks (Bányai et al., 2011; Wadehra et al., 2013)*.

The link with cognitive control is potentially illuminating for multiple reasons. Cognitive control is a meta-process that facilitates attention vigilance, context-dependent cognitive task switching, and the extraction and maintenance of process-relevant information (Banich, 2009). Each of these sub-processes, particularly attention vigilance and context-dependent process switching are relevant in the context of associated memory and retrieval. Attention mechanisms must underpin the encoding and retrieval of associative memories, and context-dependent task switching is a characteristic of alternating between modes of memory encoding and memory retrieval. While schizophrenia patients show both impairments in, and dysfunctional activation of brain regions associated with, cognitive control (Kerns et al., 2005; Snitz et al., 2006), these studies have not been conducted in the context of associative memory.

Cognitive control mechanisms are strongly associated with each of the dPFC and the dorsal anterior cingulate cortex (dACC), with precedence for ascribing potentially non-overlapping control-related attributes to each. Previous research has implicated the dPFC in processes such as the active maintenance of memoranda (Curtis and D’Esposito, 2003; Simons and Spiers, 2003), and executive control during psychological processes (Banich, 2009). Conversely, the dACC has been implicated in conflict monitoring and process-relevant attention. Therefore, it is plausible that these roles of the dPFC and dACC are expressed in differential brain network profiles in schizophrenia. Moreover, with memory encoding and memory retrieval exerting distinct demands in associative memory (Woodcock et al., 2015), it is also plausible that dPFC- and dACC-hippocampus network interactions in schizophrenia are differentially impaired in encoding and retrieval.

The focus of the present study was to characterize dysfunctional network signatures of each of the dPFC and the dACC during associative memory encoding and retrieval. The specific target brain network of interest was informed by prior research (Woodcock et al., 2015) and included the hippocampus, fusiform, parahippocampal gyrus, inferior temporal gyrus, superior parietal lobule, basal ganglia, dACC, and dPFC. The nature of the overall study design allowed us to assess for each seed (dPFC and dACC), main effects of group (schizophrenia ≠ healthy controls (HC)) and memory process (encoding and retrieval), and the interaction thereof. Our particular focus was on patterns of network hypo- or hyper-modulation by each of the dPFC and the dACC (Bakshi et al., 2011) in schizophrenia, and on the modulatory effects of each on the hippocampus during encoding and retrieval.

## Materials and Methods

### Participants

The Wayne State University Institutional Review Board approved all experimental procedures. All participants (*N* = 22) provided written informed consent prior to study involvement and were paid for their participation. HC participants (*n* = 10) were free of psychiatric or neurological conditions, with an average age of 22 years (range: 18–29 years; 5 females). Schizophrenia patients (SCZ; *n* = 12) underwent the Structured Clinical Interview for Diagnostic and Statistical Manual Disorders (First et al., 1996) and met DSM-IV criteria for schizophrenia or schizoaffective disorder. All SCZ patients were stable, early course patients (<5 year from illness onset) maintained on a regimen of atypical antipsychotics (Risperidone, Olanzapine, or Aripiprazole). SCZ patients were 26 years of age (range: 19–36 years; 3 females). Groups did not differ in age (*p* > 0.10).

### Imaging Parameters

Gradient echo fMRI signals were acquired using a 4T Bruker MedSpec system with an 8 channel head coil (TR = 3 s, TA = 2 s, TE = 30 ms, matrix = 64 × 64, slices = 24, FOV = 240 mm, voxel size = 3.75 mm × 3.75 mm × 4.0 mm, images = 288). For each subject within the same session, a high-resolution structural MRI image was axially acquired using a whole brain 3D T_1_-weighted MPRAGE sequence (TR = 2200 ms, TE = 2.56 ms, flip angle = 13°, FOV = 208 × 256 mm, voxel size = 1 mm × 1 mm × 1 mm). Head motion was minimized using foam inserts surrounding each participant’s head. Participants wore earplugs to reduce scanner noise. During each scan, experimental paradigm stimuli were projected onto a screen mounted in the scanner.

### Experimental Design

**Figure 1:**
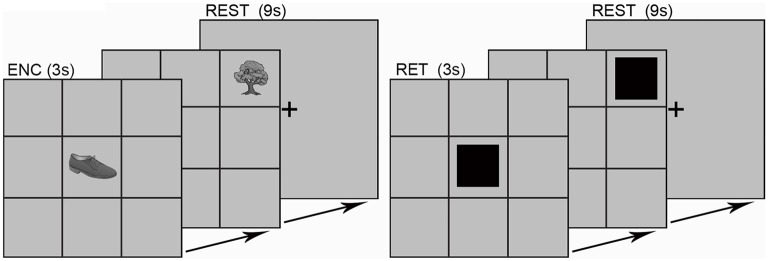
**The experimental paradigm is depicted.** Participants viewed grayscale illustrations of nine common objects with mono-syllabic names in each of the nine grid locations. Objects were presented in sequential random order during encoding (ENC; 3 s per object; 27 s total). After a brief rest (9 s; fixation cross), each location in the grid was cued in sequential random order (RET; 3 s per object; 27 s total). Participants verbalized the name of the object associated with that location. Responses were scored for accuracy using the built-in microphone/speaker system. The learning epoch sequence (ENC, REST, RET, REST, etc.) was repeated eight times (object-location pairings did not change across blocks).

Participants completed an object-location associative memory paradigm during fMRI. The paradigm (Figure 1) consisted of eight memory blocks split into epochs of encoding and location-cued retrieval (Diwadkar et al., 2008). During each encoding block, gray scale illustrations of nine common mono-syllabic objects (3 s/object; 27 s total; Snodgrass and Vanderwart, 1980) were presented in sequential random order. Each object was fixed to a unique location (that did not change across the experiment) in a nine-location (3 columns by 3 rows) spatial grid. Following a 9 s rest interval, participant’s memory of each object-location pair was tested. During each retrieval block, participants were tasked to retrieve and verbalize the identity of the object associated with the cued location (black square; 3 s/grid location; 27 s total). As is evident, in this task, encoding serves retrieval with the former memory process facilitating the latter.

Object names were monosyllabic to minimize head motion associated with verbalization. Cued-location object retrieval enforced prefrontal-driven associative memory retrieval (Desgranges et al., 1998; Allan et al., 2000). Vocal responses were scored for accuracy using the built-in microphone/speaker system. The fMRI sequence was designed to include a silent window of 1 s within the TR envelope during which responses were collected (i.e., TA < TR).

### Data Processing

MR images were preprocessed and analyzed using SPM 8 (Statistical Parametric Mapping, Wellcome Department of Imaging and Neuroscience, London, UK) using established methods for temporal (slice timing correction) followed by spatial preprocessing. For spatial pre-processing, the EPI images were manually oriented to the AC-PC line with the reorientation vector applied across the EPI image set, realigned to a reference image to correct for head movement, and co-registered to the anatomical high resolution T_1_ image. This high-resolution T_1_ image was normalized to the MNI template, with the resultant deformations subsequently applied to the co-registered EPI images for normalization. Low frequency components were removed using a low-pass filter (128 s) and images were spatially smoothed using a Gaussian filter (8 mm full-width half maximum; FWHM). An autoregressive AR(1) model was used to account for serial correlation, and regressors modeled as box-car vectors (for each of the task-related conditions: memory encoding, memory retrieval and rest) were convolved with a canonical hemodynamic reference wave form, with the six motion parameters included as effects of no interest.

### Seed Choice: dPFC and dACC

**Figure 2:**
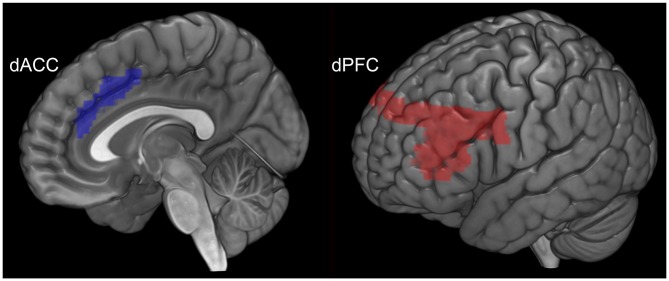
**The anatomical location and volume of each seed region is depicted.** The dACC (blue) is depicted to the left and the dPFC (red) to the right.

As previously noted, the choice of each of the dPFC and the dACC were motivated by their putatively distinct brain network profiles associated with cognitive control, including maintenance of memoranda and attention (dPFC; Simons and Spiers, 2003), and conflict monitoring and task-switching (dACC; Banich, 2009). The anatomical boundaries for each seed region (dACC [blue] and dPFC [red]) are depicted in Figure 2. To assess the network profiles of each of these regions, psychophysiological interactions (PPI) were employed. PPI allowed us to model the directional effects (i.e., seed-to-target modulation) of each of the dPFC and the dACC during each of the periods of associative memory: object-location encoding and cued retrieval. PPI is positioned between techniques of functional and maximal effective connectivity analyses (Stephan, 2004), providing a robust model for investigation of seed-based network interactions (Friston et al., 1997; Kim and Horwitz, 2008; Friston, 2011). PPIs model the response of target brain regions in terms of the interaction between a linear convolution of the physiological response of the seed region (e.g., dACC) and the contrast of interest (e.g., encoding > rest; representing the psychological context). Thus, each regressor represents the contextual effects of each seed region during each of encoding and retrieval.

For PPI modeling, time series based on an effects of interest contrast (*p* < 0.05; Woodcock et al., 2015) were extracted for a sphere (2 mm radius) centered around the statistical peak within the anatomical boundaries of each of the dPFC and dACC (Figure 2). Next, the time series for each of the dPFC and the dACC was convolved with two distinct contrasts: encoding > rest and retrieval > rest. Thus, for each participant, the resultant regressors represent contextual effects of each seed associated with each of the memorial processes of interest (encoding and retrieval). To model the hypothesized amplification effects of cognitive control, each regressor was positively-weighted (Friston et al., 1997). The strength of the interaction which is parametrically encoded in intra-subject maps was analyzed by submitting those maps to a group-level using second-level random effects analysis of variance (ANOVA).

### Statistical Design

In the second-level random effects ANOVA subject group (SCZ vs. HC) was modeled as the single independent factor, and seed (dACC vs. dPFC) and memory process (encoding vs. retrieval) modeled as non-independent factors. The associative memory network of interest was gleaned from a compendium of fMRI studies using tasks and stimuli broadly consistent with what was presently employed (Büchel et al., 1999; Ranganath et al., 2004) and included the hippocampus, fusiform, parahippocampal gyrus, inferior temporal gyrus, superior parietal lobule, basal ganglia, dACC, and dPFC. Within these target regions, statistical inference was based on cluster-level correction (*p_cluster_* < 0.05; Ward, 2000).

## Results

### Behavioral Data

**Figure 3:**
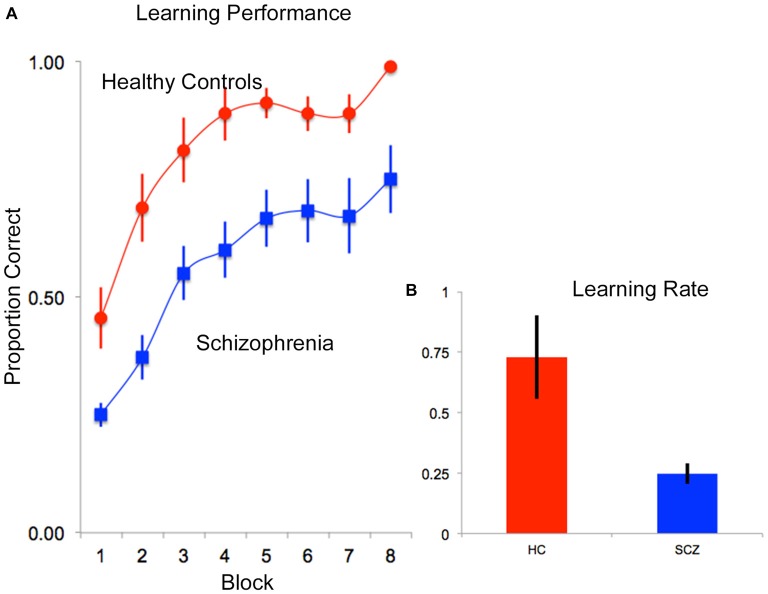
**(A)** Mean proportion correct data are depicted over time (block) for each of the HC (red) and SCZ (blue) groups. As seen, both groups exhibited negatively accelerated learning, though SCZ patients appeared to learn more slowly than HC. These observations were confirmed in an assessment of learning rate data. **(B)** Following fitting of negatively accelerated performance curves to each subjects’ performance data (proportion correct = 1−*e*^−k*epoch^; see “Results” Section) learning rates (*k*) were compared. As seen, on average, learning rate performance in patients was lower than controls. In all plots, error bars are ± SEM.

Data from one patient was not recorded on account of experimenter error, leaving data from 21 participants for the overall behavioral analyses. The behavioral data were analyzed in two ways. First, proportion correct data (ratio of correctly recalled items to total items) were analyzed in a repeated measures ANOVA with subject group (SCZ vs. HC) as the independent factor, and memory block (1–8) as the within subjects factor. Two significant effects were observed. A significant main effect of memory block (*F*_(7,133)_ = 29.75, *p* < 0.001, *MSe* = 0.022) indicated that performance improved over time (independent of subject group). In addition, a significant main effect of subject group (*F*_(1,19)_ = 8.04, *p* < 0.01, *MSe* = 0.312) indicated that memory performance was impaired in SCZ compared to HC (Figure 3A).

Second, to further assess differences in *rates of memory accumulation*, behavioral proficiency was modeled using negatively accelerated functions (proportion correct = 1−*e*^−k*epoch^) fit to memory block-wise performance data for each subject (0 ≤ proportion correct ≤ 1; Heathcote et al., 2000) in Matlab 7.1 (Mathworks, 2007). The single varying parameter, *k*, is a metric of learning rate (higher values represent more rapid learning/memory accumulation). These data were analyzed in a one-way ANOVA with subject group (HC vs. SCZ) as the single factor in the model, revealing a significant main effect of group (*F*_(1,19)_ = 7.93, *p* < 0.01, *MSe* = 0.165), indicating lower learning rates in SCZ relative to HC (Figure 3B).

### fMRI Results

**Figure 4:**
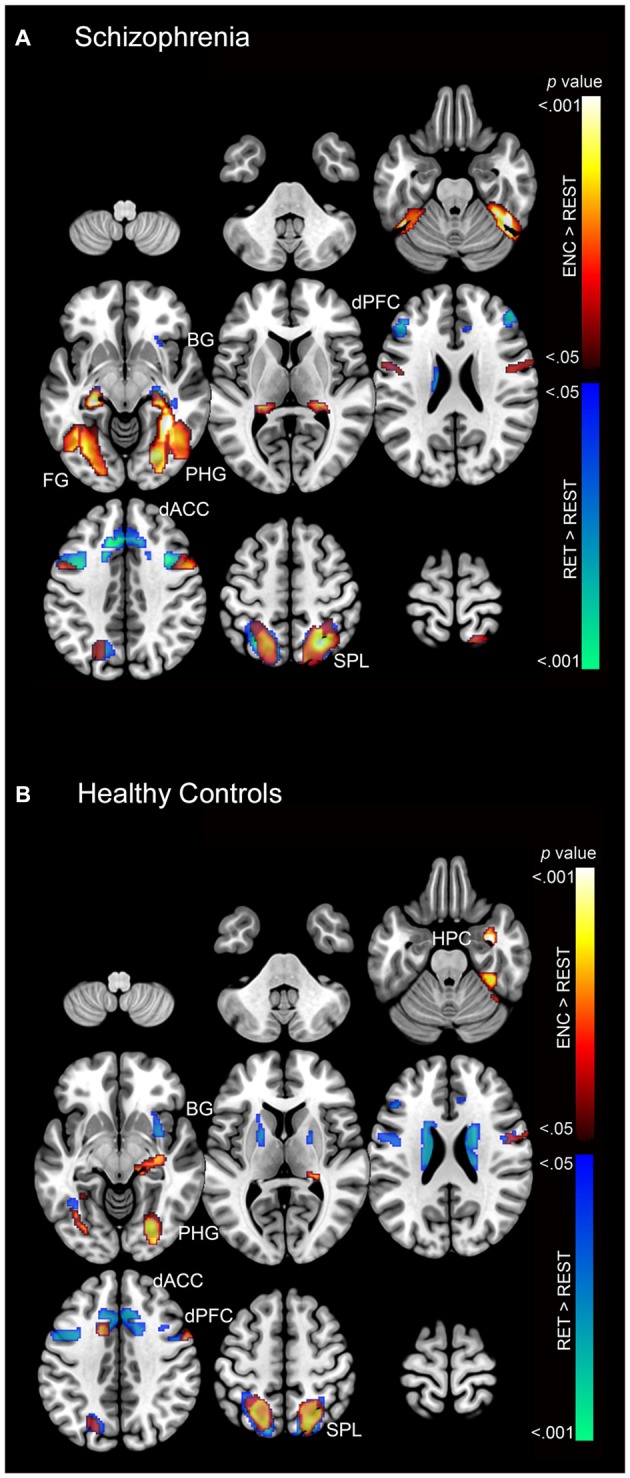
**The second-level contrasts of significant activation (cluster-level corrected; *p* < 0.05) during encoding and retrieval are depicted separately for (A) SCZ and (B) HC on a mosaic of contiguous axial slices.** For both images, significant encoding > rest clusters are depicted using a color gradient of red-yellow and retrieval > rest clusters in blue-green.

First we present the second-level contrasts showing significant activation (*p* < 0.05) during encoding and retrieval for each subject group (separately) in Figure 4A (SCZ) and Figure 4B (HC).

As these results suggest, both SCZ and HC exhibited increased activation during both encoding and retrieval relative to rest (fixation cross) in regions throughout the associative memory network. In particular, increased activation was observed in SCZ during encoding in memory (fusiform, hippocampus, and parahippocampal gyrus) and association structures (superior parietal lobule), with focal clusters observed in the dPFC. During retrieval, SCZ exhibited focal activation in prefrontal (dACC and dPFC), association (superior parietal lobule), and memory structures (fusiform, hippocampus, and parahippocampal gyrus). Conversely, HC exhibited focal activation during both encoding and retrieval across the associative memory network. HC exhibited large clusters of activation in the superior parietal lobule during both encoding and retrieval.

### PPI Results Overview

**Figure 5:**
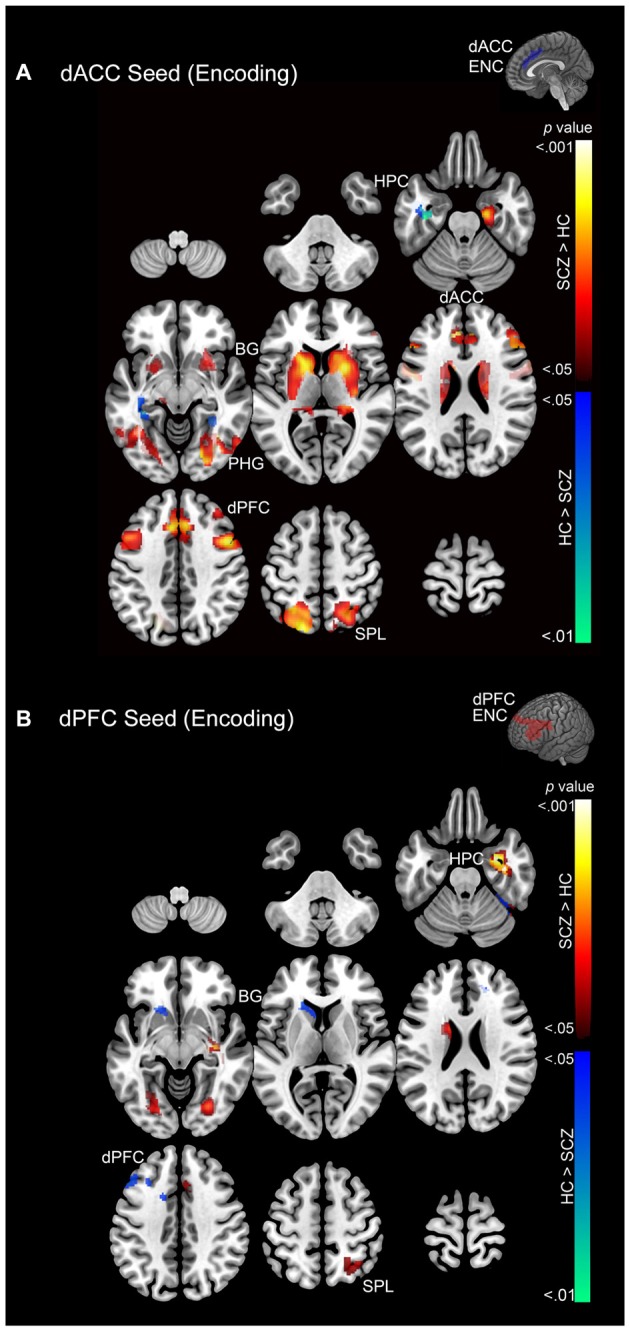
**(A)** Emanating from the dACC, significant clusters (cluster-level corrected; *p* < 0.05) of increased modulation during encoding (>rest) are depicted on a mosaic of contiguous axial slices. **(B)** Significant clusters emanating from the dPFC during encoding are depicted on a mosaic of contiguous axial slices. For both images, significant SCZ > HC clusters are depicted using a color gradient of red-yellow and HC > SCZ clusters in blue-green.

**Figure 6:**
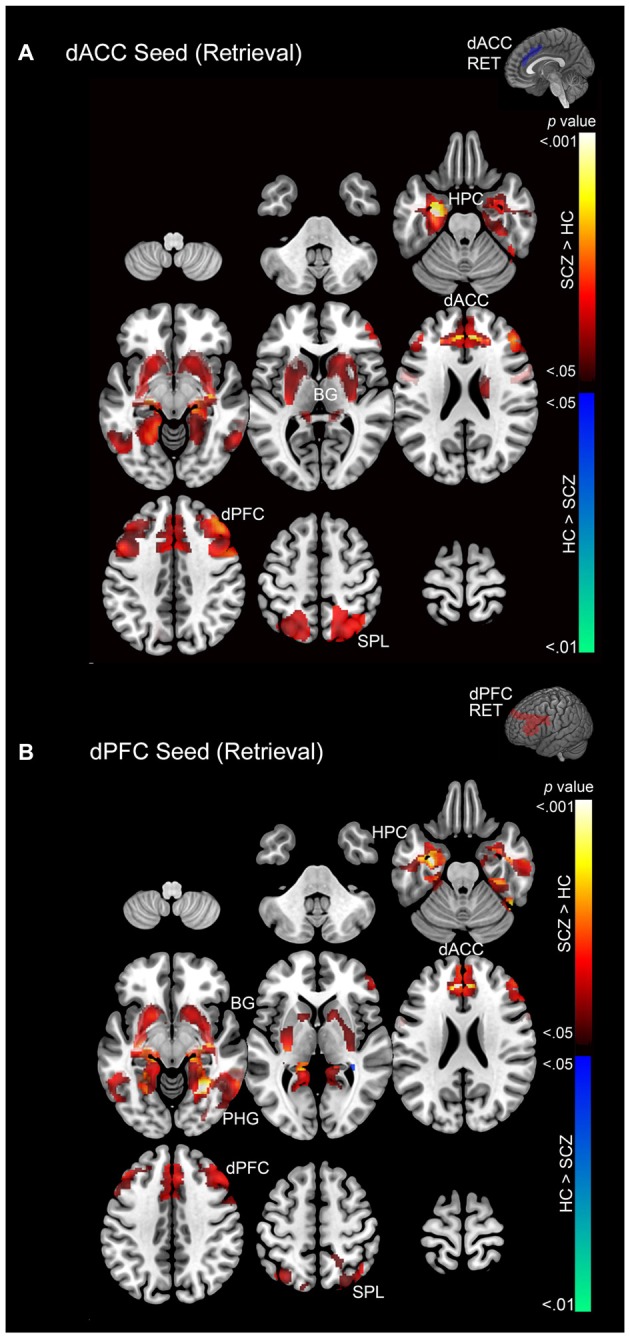
**(A)** Emanating from the dACC, significant clusters (cluster-level corrected; *p* < 0.05) of increased modulation during retrieval (> rest) are depicted on a mosaic of contiguous axial slices. **(B)** Significant clusters emanating from the dPFC during retrieval are depicted on a mosaic of contiguous axial slices. For both images, significant SCZ > HC clusters are depicted using a color gradient of red-yellow and HC > SCZ clusters in blue-green.

**Figure 7:**
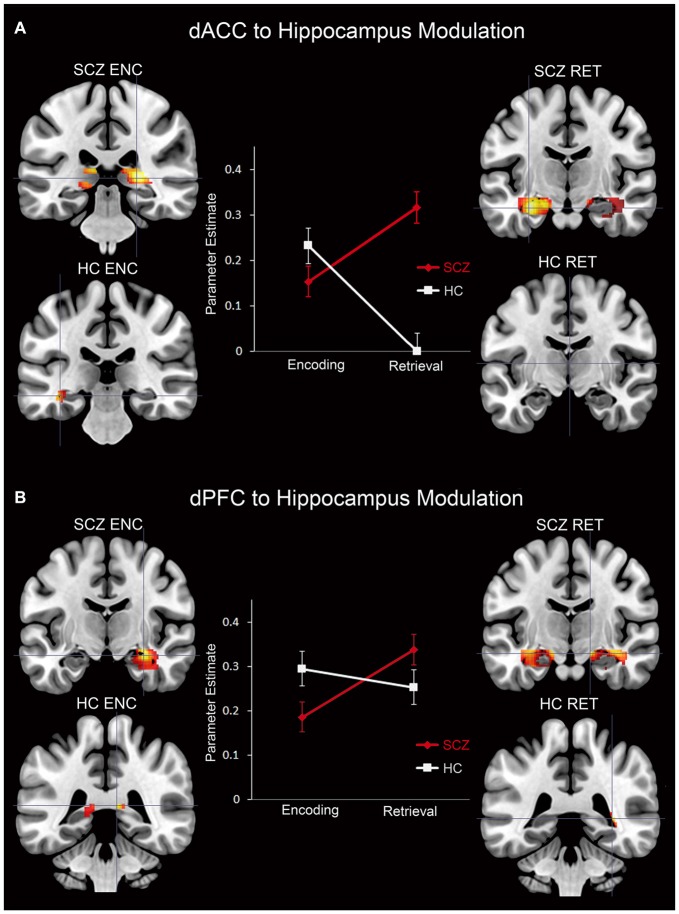
**Parameter estimates (positively-weighted) extracted from peak modulation (2 mm radius sphere) in the hippocampus for the (A) dACC and the (B) dPFC for each subject group and memory process are depicted in line graphs**. In addition, signification clusters from peak modulation in the hippocampus for each subject group and memory process are depicted on coronal slices adjacent to each graph. In the brain images, the magnitude of modulation is depicted using a color gradient of red-yellow.

**Figure 8:**
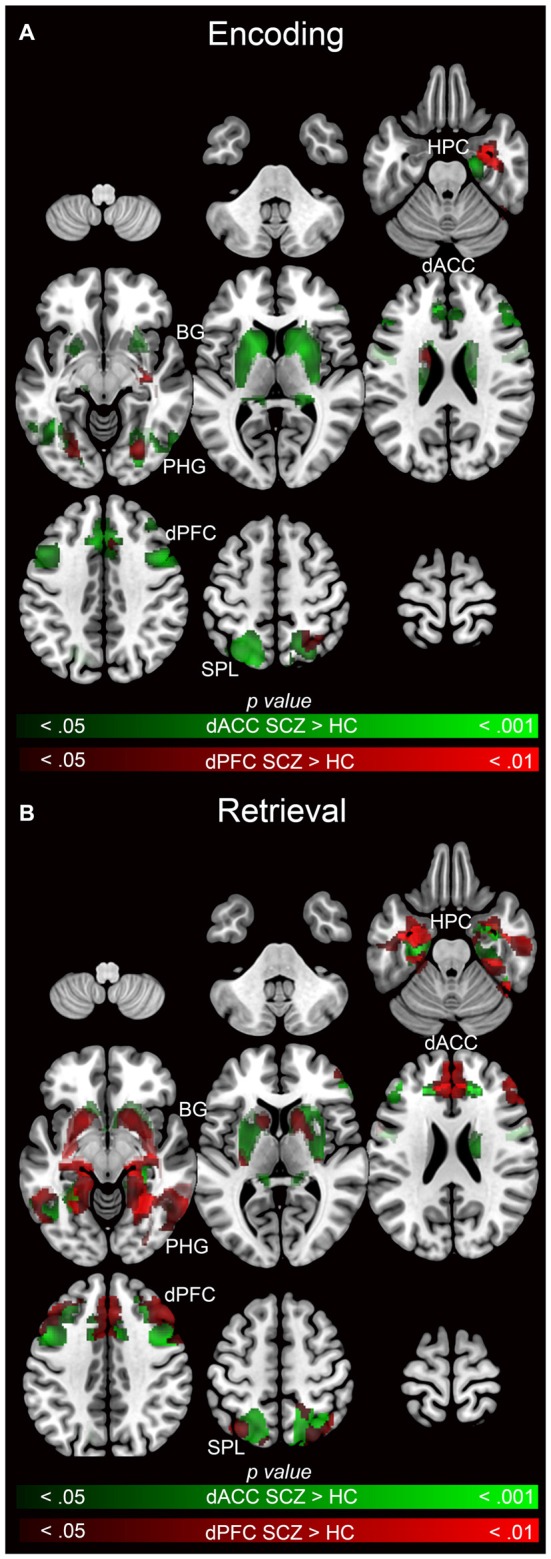
**Emanating from each the dACC (green) and dPFC (red), significant clusters (cluster-level corrected; *p* < 0.05) of increased modulation in SCZ (>HC) subjects during (A) encoding (>rest) and (B) retrieval (>rest) are depicted on mosaics of contiguous axial slices**.

Subsequent effects are devoted to explicating the PPI results. The PPI results are organized to sequentially depict group differences (SCZ ≠ HC) in modulation by each of the dPFC and the dACC for each memory process (Encoding: Figure 5 and Retrieval: Figure 6). Thus, in Table 1 and Figure 5 we depict group differences (SCZ ≠ HC) in the modulation of target brain regions during *memory encoding*. Next, in Table 2 and Figure 6 we present group differences in the modulation of target brain regions during *memory retrieval*. In Figure 7, we present evidence of a group by memory process interaction in the hippocampus. Figure 8 provides a visual summary of the relative increases in the modulatory effects of each of the dACC and the dPFC during encoding and retrieval.

**Table 1:** **This table depicts significant cluster extents (K_e_) and peak modulation observed for each of the conducted contrasts during encoding (Figures 5A,B)**.

Figure	Seed	Contrast	Region(s)	Ke	*t* value	*p* voxel	MNI (*x*, *y*, *z*)
5A	dACC	SCZ > HC encoding	L dACC	1119	5.68	< 0.001	(−6, 18, 46)
			R Fusiform gyrus	1270	5.35	< 0.001	(28, −84, −14)
			R Basal ganglia	2589	4.86	< 0.001	(22, 4, 12)
			L SPL	1483	4.82	< 0.001	(−26, −68, 40)
			R dPFC	1315	4.79	< 0.001	(48, 8, 40)
			L Basal ganglia	2261	4.52	< 0.001	(−12, 10, 12)
			L dPFC	755	4.45	< 0.001	(−50, 20, 26)
			L dACC	506	4.30	< 0.001	(−8, 40, 16)
			R Hippocampus	232	3.91	< 0.001	(20, −16, −24)
			L Fusiform gyrus	999	3.83	< 0.001	(−24, −76, −16)
			R SPL	731	3.70	< 0.001	(26, −62, 50)
			R PHG/ITG	190	3.36	< 0.001	(24, −32, 4)
			R Basal ganglia	143	2.77	< 0.01	(−12, −32, 10)
5A	dACC	HC > SCZ encoding	R PHG	59	3.10	< 0.01	(32, −46, −6)
			R SPL	14	2.73	< 0.01	(48, −50, 58)
			L PHG/Hippocampus	344	2.71	< 0.01	(−48, −4, −26)
			R dACC	33	2.05	< 0.05	(20, 34, 28)
5B	dPFC	SCZ > HC encoding	R Hippocampus	1083	3.69	< 0.001	(30, −12, −16)
			R PHG/ITG	319	3.67	< 0.001	(26, −74, −4)
			L PHG	210	3.62	< 0.001	(−22, −74, −4)
			R dACC	416	3.51	< 0.001	(2, 22, 46)
			L Basal ganglia	127	3.29	< 0.001	(−14, 0, 22)
			L dPFC	77	3.10	< 0.01	(−50, 18, 26)
			R SPL	279	2.55	< 0.01	(30, −42, 48)
5B	dPFC	HC > SCZ encoding	L dPFC	152	3.00	< 0.01	(−30, 24, 34)
			R dACC	107	2.88	< 0.01	(18, 40, 28)
			L dACC	54	2.69	< 0.01	(−18, 28, 32)
			R Fusiform gyrus	106	2.50	< 0.01	(48, −58, −22)
			L Basal ganglia	223	2.44	< 0.01	(−20, 22, −6)
			L dACC	26	2.09	< 0.05	(−16, 6, 40)

*L, Left; R, Right; PHG, parahippcampal gyrus; ITG, Inferior Temporal Gyrus; dACC, dorsal anterior cingulate cortex; dPFC, dorsal prefrontal cortex; SPL, superior parietal lobule*.

**Table 2:** **The table depicts significant cluster extents (K_e_) and peak modulation observed for each of the conducted contrasts during retrieval (Figures 6A,B)**.

Figure	Seed	Contrast	Region(s)	Ke	*t* value	*p* voxel	MNI (*x*, *y*, *z*)
6A	dACC	SCZ > HC retrieval	L Hippocampus	10802	6.68	< 0.001	(−30, −14, −20)
			R dPFC	2253	6.09	< 0.001	(40, 34, 36)
			L dACC	3165	5.29	< 0.001	(−2, 18, 30)
			L dPFC	1316	5.09	< 0.001	(−44, 38, 28)
			R SPL	1401	4.75	< 0.001	(32, −68, 60)
			L SPL	1413	4.73	< 0.001	(−22, −72, 60)
6A	dACC	HC > SCZ retrieval	n.s.	n.s.	n.s.	n.s.	n.s.
6B	dPFC	SCZ > HC retrieval	R PHG/ITG	5714	4.91	< 0.001	(56, −30, −16)
			L,R dACC	2357	4.74	< 0.001	(0, 46, 30)
			L Basal ganglia	3727	4.69	< 0.001	(−28, −20, −2)
			R dPFC	1501	4.24	< 0.001	(60, 20, 26)
			L dPFC	477	3.96	< 0.001	(−44, 30, 40)
			R SPL	484	3.88	< 0.001	(32, −70, 60)
			L PHG/ITG	381	3.64	< 0.001	(−56, −48, −6)
			L SPL	344	3.59	< 0.001	(−42, −62, 56)
6B	dPFC	HC > SCZ retrieval	R PHG/Hippocampus	22	2.39	< 0.01	(34, −40, 2)

*L, Left; R, Right; PHG, parahippcampal gyrus; ITG, Inferior Temporal Gyrus; dACC, dorsal anterior cingulate cortex; dPFC, dorsal prefrontal cortex; SPL, superior parietal lobule*.

### Memory Encoding

During memory encoding SCZ were characterized by exaggerated modulation by the dACC (Figure 5A), with increased dACC modulation observed in multiple forward visual (fusiform) and memory structures (hippocampus), association areas (superior parietal lobule), and motor and memory regions (basal ganglia). Conversely, in SCZ, dACC modulation was relatively decreased in the superior parietal lobule, parahippocampal gyrus, hippocampus, and dACC (Figure 5B).

In comparison, SCZ were characterized by exaggerated dPFC modulation in the hippocampus, parahippocampal gyrus, dACC, dPFC, basal ganglia, and superior parietal lobule, but decreased modulation of the dACC, dPFC, basal ganglia, and fusiform gyrus (Figure 5B).

### Memory Retrieval

In SCZ exaggerated modulation of targets was particularly evident during memory retrieval. The dACC exerted greater modulation of areas including the dPFC, dACC, hippocampus, and superior parietal lobule (Figure 6A) with no areas of diminished modulation. Similarly the dPFC exerted greater modulation of the parahippocampal gyrus, dPFC, dACC, basal ganglia, hippocampus, and superior parietal lobule (Figure 6B). Conversely, diminished modulation by the dPFC in SCZ relative to HC was observed in a single cluster in the parahippocampal gyrus and hippocampus (Figure 6B).

### Interaction in the Hippocampus

For each of the dACC and dPFC, an interaction between group and memory process was observed in the hippocampus (Figure 7). The PPI parameter estimates plotted in conjunction with clusters under the interaction depict the crossover interaction for each (dACC and dPFC, respectively). Specifically, relative to HC, SCZ were characterized by *decreased* modulation of the hippocampus during *encoding*, but *increased* modulation during *retrieval*. When considered against the idea that encoding serves retrieval, this interaction between memory process and group suggests a pattern of altered frontal-hippocampal interactions that may be associated with the observed associative memory deficits. In particular, reduced modulation during encoding may impair the fidelity of hippocampal memory traces, and subsequently require greater modulation when the retrieval related memory cue is generated. This is further elaborated in the “Discussion” Section.

### Visualizing Effects for Each Seed Relative to Each Other

Figure 8 facilitates direct visual comparison of each seed region during encoding (Figure 8A) and retrieval (Figure 8B), where each figure represents the relative extent of exaggerated modulation (SCZ > HC) for each memory process. During encoding, the dACC related network profiles were more dysfunctional than the dPFC, suggesting that network signatures of the dPFC were less sensitive in discriminating SCZ from HC during encoding. However, during retrieval, dysfunctional network profiles of the dACC and the dPFC were similar.

## Discussion

In this study, we contrasted the network profiles of stable, early course SCZ patients with HC subjects during an established object-location associative memory task. PPI analyses were used to model network interactions originating in the dACC and the dPFC during alternating phases of memory encoding and location-cued retrieval. The principal findings of this study were:

1. *Associative memory proficiency*. Both groups demonstrated task compliance as evidenced by the negatively-accelerated learning curves during associative memory task performance. However, learning proficiency (i.e., learning rates) and capacity (performance asymptote) were reduced in SCZ relative to HC.
2. *PPI effects during memory encoding*. In SCZ, greater dACC modulation was observed across regions of interest relative to HC. The extent of the increase in patients was greater for the dACC than the dPFC (Figure 5).
3. *PPI effects during memory retrieval*. Both the dACC and the dPFC in SCZ evinced exaggerated modulation compared to HC across the associative memory network. Conversely, HC exhibited only one significant cluster of increased modulation relative to SCZ (Figure 6).
4. *Interaction in the hippocampus*. Within this structure, SCZ were characterized by decreased modulation during encoding but increased modulation during retrieval (Figure 7).

**Figure 9:**
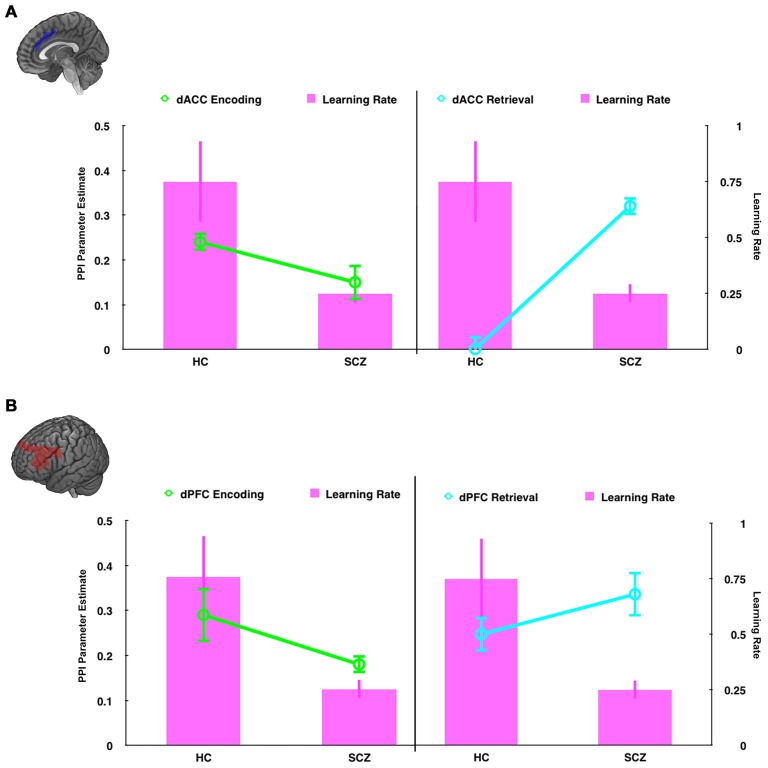
**In dual axis graphs, we plot parameter estimates associated with modulation of the hippocampus (left axis) and learning rate (right axis).** Data are plotted for the **(A)** the dACC seed and **(B)** the dPFC seed during encoding (left column, green lines ± SEM) and retrieval (right column, teal lines ± SEM). The learning rate data (magenta bars, ± SEM) are plotted in all four graphs. The figure emphasizes the relationship between learning rate and modulatory effects on the hippocampus. First, learning rate is closely related to modulatory effects during encoding. Thus, in HC hippocampal modulation and learning rates are higher than SCZ. Second, learning rate is inversely related to modulatory effects during retrieval. Thus, in HC hippocampal modulation is low, but learning rates are high, with the inverse effect observed in SCZ.

In the remainder of this manuscript, we interpret the results of the PPI analyses from a network/process perspective, and in the context of the learning proficiency results. In addition, we speculate on system’s level mechanisms that might explain the dysfunctional network interactions observed in schizophrenia and their behavioral implications (summarizing these effects in Figure 9). We conclude by noting the interpretational limitations of these analyses.

The selection of the dACC and the dPFC as seed regions was motivated by their hypothesized roles in cognitive control during and beyond associative memory function, a subject of focus in our recent work (Woodcock et al., 2015). Examination of dACC modulation is especially interesting given its established role in conflict monitoring, and its anatomical location between the frontal and motor regions (Paus, 2001; Asemi et al., 2015). Recent findings have demonstrated exaggerated dACC modulation during cognitive task performance across several psychiatric disorders, including obsessive-compulsive disorder (Diwadkar et al., 2015), and in the schizophrenia spectrum (Bakshi et al., 2011). The dPFC is implicated in executive control of psychological processes, active maintenance of memoranda during associative memory (Curtis and D’Esposito, 2003; Simons and Spiers, 2003), and is considered an “origin” of network signals during cued memory retrieval (Miller and D’Esposito, 2012). PPI analyses facilitated examination of directed seed-to-target network relationships as a function of control-related demand during each phase of associative memory.

The observed effects establish two broad results. First, in SCZ, dysfunctional network profiles were apparent during both encoding and retrieval, *but were particularly pronounced during the latter* (Figures 5, 6). Second, in the hippocampus, we observed a compelling crossover interaction between memory process and subject group, whereby in schizophrenia, modulation of the hippocampus during encoding by each of the dACC and the dPFC was diminished, only to be *increased* during subsequent retrieval (Figure 7).

The pattern of widespread and exaggerated modulation from the dACC in SCZ suggests that the structure’s role in conflict monitoring is particularly salient in this disease state. A plausible systems-level explanation is that in schizophrenia, the dACC is forced to execute cognitive control of task-related error monitoring by significantly amplifying network wide activity during encoding, a pattern that did not generalize to the dPFC (note the relative lack of overlapping clusters in Figure 8A). In comparison to encoding, in schizophrenia retrieval was characterized by exaggerated network modulation by both the dACC and the dFPC (note the pattern of overlapping clusters in Figure 8B).

Retrieval is a particularly demanding phase of associative memory function, requiring cued search of existing memory traces, and relies on the integrity of these encoded memory traces. Our data indicate diminished behavioral performance in patients, implying a plausible lack of network integrity during encoding. The substantively exaggerated patterns of network modulation emanating from the dACC during subsequent retrieval imply exaggerated control-related demand associated with error and conflict monitoring during retrieval of relatively poorly encoded traces. Retrieval cues are hypothesized to originate in the dPFC (Simons and Spiers, 2003), and thus, network signatures of the dPFC during retrieval are of particular interest. The dPFC exerted increased modulation in medial temporal structures (e.g., hippocampus, parahippocampal gyrus), striatum, and areas associated with spatial encoding (e.g., superior parietal lobule). The pattern of modulation in regions of interest suggests a network effect of pathology expressed at the level of macroscopic network interactions. However, the pathology is not merely characterized in exaggerated modulation, but is expressed additionally in subtle network dysfunction.

Schizophrenia patients were also characterized by reduced modulation of the hippocampus during encoding, but greater modulation during retrieval. As Figure 9 depicts, this effect parallels the behavioral results. We propose that the reduced frontal modulation of the hippocampus during encoding impairs the fidelity of memory traces, resulting in increased modulation during retrieval as a compensatory mechanism. The relative specificity of the interaction within the hippocampus, coupled with the widespread effects of retrieval related hyper-modulation across the network suggest two complementary expressions of dysfunction in brain network interactions in schizophrenia that can be tentatively associated with separate mechanisms; a pathologic signature of mechanistic complexity that is ubiquitously represented in brain network function (Pessoa, 2014). Thus, exaggerated modulation across the network may represent inefficiently expressed mechanisms of cognitive control distributed across the associative memory network (Bakshi et al., 2011). However, the specific interaction within the hippocampus may represent a focal network deficit, highly circumscribed to frontal-cingulate-hippocampal network processes (Heckers, 2001; Brambilla et al., 2007, 2011), and proximate to the observed learning deficits in schizophrenia.

### Limitations

Several experimental limitations are worth noting. Our PPI analyses, conducted within the framework of a block design, do not permit assessment of processes that might be specific to the successful (or unsuccessful) retrieval of individual memoranda. Moreover, PPIs constitute relatively simplistic models of brain network interactions (Stephan, 2004), limited in terms of interpretational utility and sensitivity to the “hidden” brain states that mediate the emergence of fMRI signals (Friston et al., 2013). Their utility is noted for examining more specific process-oriented hypotheses that are tied to directional effects between pairs of brain regions.

We have suggested that the exaggerated nature of frontal-hippocampal modulation in schizophrenia patients reflects aspects of network inefficiency. While the construct of inefficiency has been criticized as being unconstrained (Poldrack, 2015), in the present study, it is grounded in concurrently acquired and well-characterized behavioral data (learning proficiency results). These data suggest that SCZ patients were highly compliant with task instructions (as they evinced classic negatively accelerated learning), but not as effective (reduced memory capacity and proficiency). In that sense, the exaggerated network interactions described herein as “inefficient” are underpinned by *diminished* behavioral proficiency.

### Summary

The investigation of brain network profiles may improve our understanding of network dysfunction in psychiatric conditions. Interpretation of fMRI network analyses is limited (primarily to interactions of cumulative “neural” signals at the macroscopic scale), and the relationship to neurochemical or molecular bases of the disease state is unclear. However, the characterization of network profiles may reveal systems-level mechanisms associated with psychiatric diagnoses, disease progression, and treatment response. Our findings illustrate plausible network bases that may underlie impaired associative memory performance in schizophrenia. Specifically, diminished frontal-hippocampal modulation during memory encoding may result in sub-optimal memory trace fidelity, which in turn, results in amplified network effects during cued memory retrieval. Inferring “hidden states” from observed and modeled fMRI data is a significant challenge (Friston et al., 2012, 2013). Nevertheless, systems-level analyses, such as presented here, can facilitate discovery of macroscopic network dysfunction in schizophrenia, motivating the search for underlying molecular and/or neural bases.

## Author Contributions

EAW conducted half of the data analyses, produced the figures and tables, and drafted the manuscript. SW conducted the other half of the data analyses and edited the manuscript. VAD collected the data presented herein, conceptualized the study design, and edited the manuscript. EAW, SW and VAD approved the final version of the manuscript for publication.

## Conflict of Interest Statement

The authors declare that the research was conducted in the absence of any commercial or financial relationships that could be construed as a potential conflict of interest.
